# Novel Insights into G0S2 as a Central Regulator of Lipid Metabolism and Its Implications for Meat Quality

**DOI:** 10.3390/ani16101467

**Published:** 2026-05-10

**Authors:** Li Han, Hongkun Li, Jiajie Ouyang, Chunru Lu, Tao Jing, Haiqing Gan, Jie Yin, Qiyu Tian, Xingguo Huang

**Affiliations:** 1 College of Animal Science and Technology, Hunan Agricultural University, Changsha 410128, China; hl68497075@126.com (L.H.); lhko328@163.com (H.L.); 17674911120@163.com (J.O.); luchun_ru@163.com (C.L.); 19186855851@163.com (T.J.); ghq0619123@163.com (H.G.); yinjie2014@126.com (J.Y.); 2 College of Biology and Food Engineering, Fuyang Normal University, Fuyang 236041, China; 3 Department of Agriculture and Rural Affairs of Hunan Province, Changsha 410005, China

**Keywords:** G0S2, lipid metabolism, lipolysis, adipogenesis, intramuscular fat, meat quality

## Abstract

The increasing demand for premium-quality meat has intensified interest in the molecular regulators that govern lipid deposition and sensory quality. Lipid metabolism, encompassing synthesis, oxidation, and storage, represents a key biological process affecting intramuscular fat content, tenderness, and flavor. G0/G1 Switch Gene 2 (*G0S2*) is widely recognized as an endogenous, non-competitive inhibitor of adipose triglyceride lipase. By inhibiting this key lipase, *G0S2* restrains triglyceride hydrolysis and helps preserve lipid storage. Recent studies further suggest that *G0S2* participates in adipocyte differentiation, mitochondrial regulation, apoptosis, and inflammatory signaling. Together, these findings indicate that *G0S2* functions not only in lipolysis control but also as a multifunctional regulator of energy metabolism and cellular homeostasis. Despite its pleiotropic roles, which position *G0S2* as a key integrator of lipid metabolism and cellular signaling networks, the specific roles and regulatory mechanisms by which *G0S2* influences lipid metabolism and meat quality remain incompletely understood. This review summarized recent advances in *G0S2*-mediated lipid metabolism with emphasis on its regulatory network in livestock species. The physiological mechanisms by which *G0S2* modulated lipolysis, lipid deposition, and intramuscular adipogenesis were discussed, along with nutritional, hormonal, and epigenetic factors controlling its expression. Moreover, its functional implications for meat quality improvement, marbling formation, and feed efficiency were highlighted. Understanding the molecular and regulatory features of *G0S2* provided a foundation for developing genetic and nutritional strategies to optimize lipid utilization and enhance meat quality in modern animal production systems.

## Simple Summary

Meat quality, particularly its tenderness, juiciness, and flavor, is increasingly important to consumers and is largely influenced by intramuscular fat. This review focuses on a key biological regulator of fat metabolism called G0/G1 Switch Gene 2 (*G0S2*). Acting as a natural brake on fat breakdown, *G0S2* helps control the accumulation of intramuscular fat by inhibiting an enzyme responsible for mobilizing stored fat. Higher activity of *G0S2* promotes marbling, which enhances meat quality. The expression of the *G0S2* gene is influenced by factors like nutrition and genetics, making it a promising target for improving livestock. Understanding how to manage *G0S2* through breeding or feeding strategies offers a direct pathway to tailor fat deposition in animals, thereby enhancing meat quality for the consumer while maintaining efficient production. This research bridges fundamental biology with practical applications in sustainable animal agriculture.

## 1. Introduction

In recent years, improvement of meat quality has become a priority in modern livestock production as consumers increasingly valued tenderness, flavor, and nutritional value rather than carcass yield. Among the biological factors affecting these sensory traits, lipid metabolism is recognized as a key determinant because it governed intramuscular fat (IMF) accumulation, fatty acid composition, and postmortem biochemical changes [1]. IMF contributes to juiciness, mouthfeel, and flavor retention, and it also influences meat texture through its impact on muscle fiber structure and water-holding capacity. Understanding the molecular mechanisms of lipid metabolism was therefore critical for improving both production efficiency and product quality. Lipid metabolism encompasses several interconnected processes, including fatty acid uptake, de novo synthesis, β-oxidation, storage, and lipolysis, the mobilization of stored triglycerides (TG) into free fatty acids (FFA) [2]. Among these, lipolysis is the first and rate-limiting step of lipid mobilization and plays a decisive role in maintaining energy balance [3]. Adipose triglyceride lipase (*ATGL*), encoded by the patatin-like phospholipase domain containing 2 (PNPLA2) gene, catalyzes the initial step of TG hydrolysis into diacylglycerol and FFA, making it the primary determinant of lipolytic rate [4]. The discovery of G0/G1 Switch Gene 2 (*G0S2*) as an endogenous, noncompetitive inhibitor of ATGL established a direct molecular link between lipid turnover and energy balance [5,6]. By directly binding to the hydrophobic domain of *ATGL*, *G0S2* restricted excessive TG hydrolysis and maintained lipid homeostasis, thereby protecting cells from lipotoxic damage [7,8,9]. This regulatory interaction positioned *G0S2* as an important control point for lipid catabolism; however, its downstream phenotypic consequences appear to be context-dependent.

Originally, *G0S2* was identified as a cell cycle–related gene that was upregulated during the transition from the quiescent (G0) to the proliferative (G1) phase in human lymphocytes [10]. Subsequent studies revealed its major role as a suppressor of ATGL activity and a key regulator of lipid and energy balance [11,12,13]. *G0S2* localized to multiple organelles, including the cytoplasm, endoplasmic reticulum (ER), mitochondria, and lipid droplets (LDs), indicating that it may participate in diverse ATGL-dependent and ATGL-independent processes such as proliferation, differentiation, mitochondrial regulation, and inflammatory signaling, all of which contributed to tissue development and metabolic efficiency [14,15,16,17,18]. Comparative expression analyses across species showed that *G0S2* was highly conserved and abundantly expressed in metabolic tissues such as adipose, liver, and skeletal muscle [19]. These tissue-specific patterns suggested that *G0S2* functions were closely associated with physiological adaptation and production traits. In livestock, variation in *G0S2* expression or activity was strongly linked to differences in IMF content and marbling, two major determinants of meat quality [20,21,22,23]. Elevated *G0S2* levels favored lipid deposition within muscle fibers, while reduced expression promoted lipolysis and energy mobilization. Modulating *G0S2* expression thus offered a potential strategy to adjust fat deposition and improve meat quality, but its biological meaning must be interpreted within the local tissue context. Furthermore, *G0S2* transcription was dynamically influenced by nutritional status, hormonal regulation, and environmental factors [24,25,26], supporting its role as a metabolic sensor that integrated systemic lipid fluxes. Given its involvement in coordinating energy storage and utilization, *G0S2* emerged as a promising molecular target for genetic and nutritional interventions aimed at enhancing meat quality and metabolic efficiency in livestock.

Although substantial progress had been made, the mechanisms by which *G0S2* integrated lipid metabolism, cellular signaling, and meat quality remained incompletely understood. Key questions persisted regarding how *G0S2* exerted its multiple actions across metabolic networks, how different stimuli shaped its transcriptional and post-translational regulation, and how these mechanisms could be exploited to improve meat quality. In particular, several aspects of *G0S2* function appeared to be highly context-dependent. Reported effects on mitochondrial activity, inflammatory signaling, apoptosis, and tissue lipid accumulation were not always consistent across different species, tissues, and experimental models. Therefore, this review summarized the current understanding of *G0S2* biology while also considering divergent findings reported in different biological settings. Particular emphasis was placed on distinguishing the role of *G0S2* in lipolysis inhibition from its potential involvement in adipogenesis, as well as on its interaction with ATGL and other lipolytic proteins, its context-dependent roles in mitochondrial function and apoptosis, and the relevance of these mechanisms to intramuscular fat deposition and meat quality in livestock.

## 2. Literature Search Strategy

For this review, we searched the PubMed database using the combined terms “*G0S2*”, “G0/G1 switch gene 2”, “*ATGL*”, “*PNPLA2*”, “lipolysis”, “lipid metabolism”, “adipogenesis”, “intramuscular fat”, “IMF”, “marbling”, “meat quality”, “pig”, “porcine”, “cattle”, “bovine”, “chicken”, “poultry”, and “livestock”. The search strategy was based on the following Boolean expression: ((*G0S2* [Title/Abstract]) OR (“G0/G1 switch gene 2” [Title/Abstract])) AND ((*ATGL* [Title/Abstract]) OR (*PNPLA2* [Title/Abstract]) OR (lipolysis [Title/Abstract]) OR (“lipid metabolism” [Title/Abstract]) OR (adipogenesis [Title/Abstract]) OR (“intramuscular fat” [Title/Abstract]) OR (IMF [Title/Abstract]) OR (marbling [Title/Abstract]) OR (“meat quality” [Title/Abstract]) OR (pig [Title/Abstract]) OR (porcine [Title/Abstract]) OR (cattle [Title/Abstract]) OR (bovine [Title/Abstract]) OR (chicken [Title/Abstract]) OR (poultry [Title/Abstract]) OR (livestock [Title/Abstract])). The search covered the period from 1 January 1991 to 30 July 2025. After identifying relevant and accessible manuscripts, we examined the references cited in each selected article and included additional studies that met our inclusion criteria. Studies were included if they reported information on *G0S2* expression, regulation, or function in relation to lipid metabolism, adipogenesis, mitochondrial function, apoptosis, inflammatory signaling, intramuscular fat deposition, marbling, or meat quality. Studies that did not meet these criteria were excluded. The selected studies were then grouped according to the major topics addressed in this review, including *G0S2*-mediated regulation of lipolysis, multifunctional roles in cellular homeostasis, tissue-specific metabolic functions, regulatory factors controlling *G0S2* expression, and its implications for meat quality in livestock.

## 3. Dynamic Regulation of Lipolysis and Lipid Deposition by G0S2

TG hydrolysis proceeded through sequential enzymatic reactions involving three major lipases: *ATGL*, hormone-sensitive lipase (*HSL*), and monoglyceride lipase [27,28,29]. However, uncontrolled or excessive lipolysis led to lipid depletion, oxidative stress, and lipotoxicity [30], thereby compromising muscle cell integrity and meat quality. *G0S2* was identified as a potent and specific inhibitor of *ATGL* [4,5], a 54 kDa hydrolase belonging to the patatin-like phospholipase domain-containing protein (PNPLA) family [30]. Importantly, *G0S2* selectively inhibited ATGL activity without affecting the activities of *HSL*, monoglyceride lipase, or other patatin-like domain-containing enzymes, including *PNPLA6* and *PNPLA7*, thus preserving lipid storage [31,32]. Structural studies revealed that the inhibitory domain of *G0S2* was localized to a major hydrophobic region (Lys20-Ala52), particularly residues 20–44, which directly bound the *PNPLA2* domain of *ATGL* [16,32]. This binding sterically restricted substrate access to the catalytic site, and inhibited hydrolytic activity in a dose-dependent and non-competitive manner [5,6,33]. Moreover, synthetic peptides mimicking this *G0S2* segment reproduced its inhibitory effects in vitro, confirming the minimal region required for *ATGL* suppression [32], further supporting that this sequence forms the minimal inhibitory domain.

ATGL activity was further modulated by its coactivator, comparative gene identification-58 (*CGI-58*, also known as α/β-hydrolase domain-containing protein 5, ABHD5), which enhanced lipolysis through a non-competing mechanism relative to G0S2 [33,34,35]. *CGI-58* facilitates the recruitment and activation of *ATGL* and other members of the PNPLA family, forming an essential co-regulatory complex for TG catabolism [30,36]. Under basal conditions, *CGI-58* was sequestered by perilipin-1 (*PLIN1*) on the surface of LDs. Upon lipolytic stimulation, hormonal signals activated protein kinase A (PKA), which phosphorylated *PLIN1*, releasing *CGI-58* to activate *ATGL* and promoted its localization to LDs, and promote lipolysis [19]. Conversely, *G0S2* acted as a counterbalance by directly binding *ATGL* and suppressing *ATGL*’s hydrolysis activity, establishing a finely tuned equilibrium between lipid mobilization and storage. Interestingly, *G0S2* also modulated the subcellular localization of *ATGL*. While *CGI-58* promoted *ATGL* association with LDs, *G0S2* binding could retain *ATGL* in the cytoplasm or alter its droplet localization depending on the cellular context [37]. Paradoxically, *G0S2* rescued the localization of C-terminally truncated ATGL mutants, implying that *G0S2* not only affected enzymatic activity but also influenced LDs dynamics [37]. The opposing yet complementary actions of *G0S2* and *CGI-58* established a dual-control mechanism ensuring lipid homeostasis under variable physiological states.

**Figure 1:**
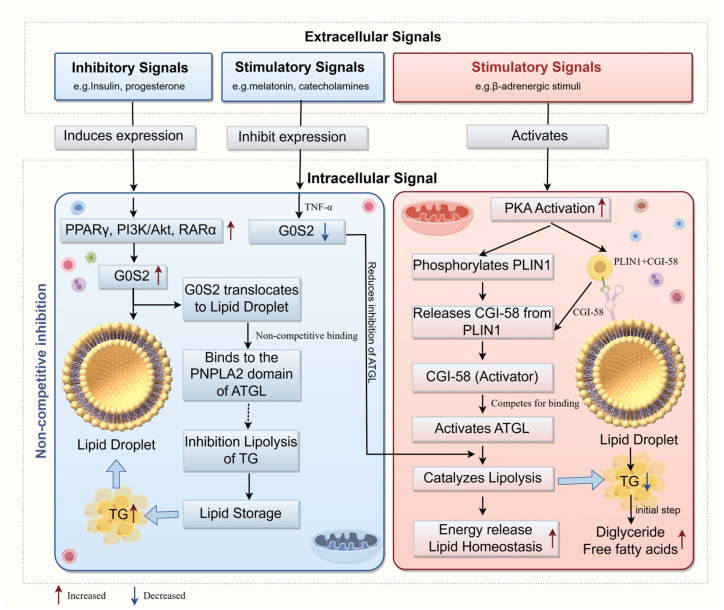
Regulatory circuit of lipolysis mediated by *G0S2* and *CGI-58*. Catecholamine-activated PKA phosphorylates perilipin and releases *CGI-58* to activate *ATGL*. Anti-lipolytic signals induce *G0S2* expression, which binds *ATGL* and inhibits hydrolysis. The non-competitive interplay of G0S2 and *CGI-58* determines lipid turnover and intramuscular fat deposition (by Figdraw2.0).

The regulatory interplay between *G0S2* and *ATGL* represented a key determinant of the balance between lipolysis and lipid storage (Figure 1), which directly influenced fat deposition in muscle and adipose tissue, a key factor in meat quality. Excessive *ATGL* activity accelerated TG hydrolysis, reduced IMF content and impaired marbling, whereas elevated *G0S2* expression restricted lipolysis, facilitated lipid accumulation and potentially enhanced meat quality. Therefore, elucidating the molecular mechanism by which *G0S2* inhibited *ATGL* provided a valuable framework for manipulating fat deposition and improving meat quality through genetic or nutritional interventions in livestock production. Nevertheless, the biological role of *G0S2* could not be fully explained by *ATGL* inhibition alone. Emerging evidence indicated that *G0S2* also participated in the regulation of proliferation, differentiation, mitochondrial function, apoptosis, and inflammatory signaling, suggesting that its contribution to tissue lipid accretion and meat quality extends beyond canonical lipolytic control.

## 4. Multifunctional Roles of *G0S2* in Cellular Homeostasis

Lipid metabolism was closely associated with essential cellular processes including signal transduction, proliferation, differentiation, and apoptosis, maintaining tissue homeostasis [3]. Beyond its well-known inhibitory role in lipolysis, *G0S2* acted as a multifunctional regulator in diverse biological pathways, with its actions varying across tissues and species [10,38]. In adipocytes, *G0S2* promoted terminal differentiation and LDs formation [39], whereas under oxidative stress it limited apoptosis and preserved mitochondrial integrity, potentially improving postmortem meat quality [40]. *G0S2* exhibited broad subcellular localization, including the cytoplasm, ER, mitochondria, and LDs [4,15]. Its N-terminal domain anchored to the cytoplasm and the C-terminal domain extending into the ER lumen, interacted with ER proteins regulating lipid synthesis and organelle signaling [41]. During metabolic stress, fasting-induced ER activation of c-AMP responsive element binding protein H (*CREBH*) and peroxisome proliferator-activated receptor α (*PPARα*) jointly increased hepatic G0S2 expression, thereby stabilizing triglyceride homeostasis [42]. *G0S2* also colocalized with mitochondrial and endosomal markers, suggesting a role in inter-organelle lipid communication [43]. Collectively, *G0S2* acted as a signaling hub connecting organelle crosstalk and energy metabolism.

### 4.1. Regulation of Cell Proliferation

*G0S2* generally restrained cell proliferation by halting the cell cycle at G0/G1. This inhibition occured through multiple mechanisms, including repression of pro-proliferative signaling pathways, interference with nucleolar protein trafficking, and modulation of cellular metabolism. It inhibited the PI3K/mTOR and MYC signaling pathways [9,44] and interacted with nucleolin to prevent its nuclear trafficking, maintaining cells in a quiescent state [43,45]. High *G0S2* levels preserved hematopoietic and adipose stem cell quiescence, while its silencing triggered abnormal proliferation. *G0S2* overexpression or demethylation restored its expression and suppressed cell proliferation by binding to the arginine–glycine–glycine domain of nucleolin, causing its cytoplasmic retention and loss of nucleolar function. Subcellular localization analyses confirmed that nucleolin was perinuclear in *G0S2*-high stem cells but nucleolar in *G0S2*-deficient progenitors [43,46]. In immune cells, *G0S2* limited mitochondrial oxidative phosphorylation (OXPHOS) in naïve CD8^+^ T cells, maintaining a quiescent metabolic state [45]. Similarly, in adipose tissue, G0S2 expression inversely correlated with preadipocyte proliferation. In pigs, G0S2 levels were low during fetal and neonatal stages, periods of rapid proliferation, but higher expression levels in adult adipocytes when adipocytes become post-mitotic and lipid-filled [47]. In chickens, *G0S2* deletion enhanced preadipocyte proliferation and DNA synthesis, while reduced G1-phase cells compared with wild type cells, indicating that *G0S2* suppressed adipocyte number expansion [48]. As adipocyte number influenced IMF content, these data suggested that *G0S2* might modulate meat quality indirectly through proliferation control. Thus, *G0S2* likely contributed to meat quality by modulating cell cycle arrest and adipose tissue expansion.

### 4.2. Regulation of Cell Differentiation

*G0S2* served as a positive regulator of adipocyte differentiation, linking growth arrest to terminal adipogenesis and lipid droplet formation. During embryonic development, *G0S2* expression increases in differentiated liver and muscle cells that have exited the cell cycle [10]. In 3T3-L1 fibroblasts, transient *G0S2* induction during the G1 phase preceded adipogenic differentiation, marking a critical transition from proliferation to lipid accumulation [10,41]. Similarly, in developing adipose tissue, high *G0S2* levels in mature adipocytes correlated with cell-cycle exit and differentiation [47].

Mechanistically, *G0S2* acted downstream of *PPARγ* and CCAAT/enhancer-binding protein α (*C/EBPα*) promote adipocyte differentiation. Its induction during early adipogenesis paralleled the expression of late markers such as adaptor protein 2 and glycerol-3-phosphate dehydrogenase [41]. A conserved PPAR response element in the *G0S2* promoter (−2.2 to −1 kb) mediated direct transcriptional activation by *PPARγ*. Overexpression of *G0S2* enhanced *PPARγ* and *C/EBPα* expression and lipid accumulation, and increased LDs formation in 3T3-L1 cells, whereas knockdown suppressed differentiation, reduced expression of key regulators, induced apoptosis, and diminished lipid accumulation [39]. Similar results were observed in chicken preadipocytes, where *G0S2* knockout reduced Fatty Acid Synthase (*FASN*) and *PLIN1* expression and decreased LDs formation [48]. In vivo, *G0S2*-deficient mice exhibited smaller adipocytes and lower fat mass, confirming its role in terminal differentiation [39]. Therefore, available evidence supported the involvement of *G0S2* in adipocyte maturation and LD formation; however, this did not necessarily indicate that all *G0S2*-associated fat accumulation resulted from adipogenesis alone. In livestock tissues, the relative contribution of adipocyte differentiation and reduced lipolysis likely varied according to developmental stage, adipose depot, and the local metabolic environment of skeletal muscle.

### 4.3. Mitochondrial Function and Apoptosis

Beyond proliferation and differentiation, *G0S2* also regulated mitochondrial function and apoptosis in a highly context-dependent manner. Mitochondria played a central role in reactive oxygen species (ROS) generation, regulation of cellular energy metabolism, and apoptosis [49,50]. *G0S2* modulated mitochondrial activity and apoptosis in a cell-type-dependent manner. When localized to mitochondria, *G0S2* interacted with FOF1-ATP synthase to enhance OXPHOS and ATP production, reducing ROS accumulation and improving survival under hypoxia in zebrafish and cardiomyocyte models, thereby improving mitochondrial function [51,52]. Its stability was regulated by proteasomal degradation via the RNF126/BAG6 complex, and inhibiting this process increased ATP production under hypoxia [53]. Conversely, in CD8^+^ T cells, it maintained metabolic quiescence by suppressing OXPHOS [45]. These apparently opposing findings suggested that the effect of *G0S2* on mitochondrial function was not uniform, but instead varied with cell lineage and energetic demand, such that *G0S2* might either promote ATP-generating activity or help maintain metabolic quiescence. *G0S2* also exhibited diverse roles in inflammatory responses, exerting both anti-inflammatory and pro-inflammatory effects in a tissue-specific manner. In 3T3-L1 adipocytes, Tumor necrosis factor-alpha (TNF-α) suppressed *G0S2* expression via *PPARγ* inhibition, stimulating lipolysis and potentially exacerbating metabolic inflammation [54]. In the 5/6 nephrectomy mouse model, signal transducer and activator of transcription 5B directly bound to the promoter region of *G0S2* and enhanced its transcription. The upregulation of *G0S2* in turn functioned as a co-activator for p65, promoting the transcription of pro-inflammatory genes such as chemoattractant ligand 2 and thereby exacerbating renal inflammation. Knockdown or inhibition of *G0S2* attenuated this response [13]. Accordingly, the pro-inflammatory and anti-inflammatory effects of *G0S2* needed to be interpreted within the context of tissue type, upstream signaling, and cell type, rather than being regarded as fixed intrinsic properties of the protein.

*G0S2* also exerted both pro- and anti-apoptotic functions, which were determined by cellular context and upstream signaling. Through a hydrophobic domain (Leu33–Gln67), it bound to Bcl-2 and disrupted protective Bcl-2/Bax heterodimer formation and controlling mitochondrial membrane permeability, promoting cytochrome c release and mitochondrial apoptosis, acting as a novel pro-apoptotic factor induced by TNF-α via the NF-κB pathway [17]. In macrophages, metformin upregulated *G0S2* expression, thereby inhibiting Bcl-2 and promoting apoptosis, particularly in pro-inflammatory M1 subsets, thus contributing to its anti-inflammatory effects [44,55]. Similarly, ammonia exposure in chickens upregulated *G0S2* in the jejunum, activating the mitochondrial apoptosis pathway via a lncRNA-miR-205a-G0S2 axis [56]. These findings suggested that *G0S2* mediated the pro-apoptotic actions of certain pharmacological or environmental stimuli. However, under oxidative stress, *G0S2* overexpression alleviated oxidized low-density lipoprotein (ox-LDL)-induced damage by reducing ROS production, maintaining mitochondrial membrane potential, preventing cytochrome c release, and inhibited caspase activation via the AMPK signaling pathway and upregulation of antioxidant mediators, displayed a protective, anti-apoptotic role [57,58]. In adipocytes, *G0S2* also exerted ATGL-independent anti-apoptotic effects during terminal differentiation. Depletion of *G0S2* induced apoptosis irrespective of *ATGL* expression, as shown by increased caspase-3 activation in double knockdown experiments of *G0S2* and *ATGL* [39]. These findings indicate that *G0S2* can either promote or restrain apoptosis depending on metabolic context, and that at least part of its anti-apoptotic activity during adipocyte differentiation is *ATGL*-independent.

**Figure 2:**
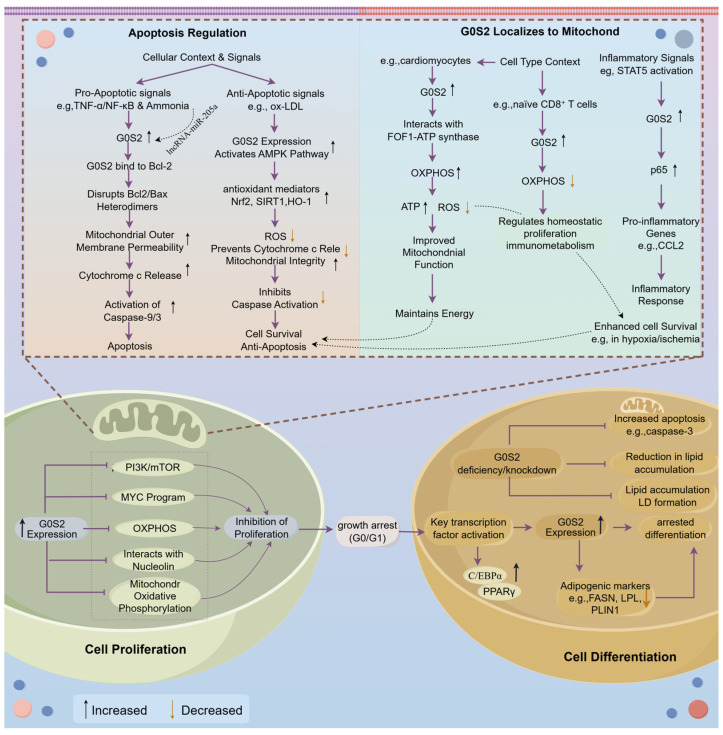
Multifunctional roles of *G0S2* in cellular homeostasis. *G0S2* suppressed proliferation via MYC and PI3K/mTOR inhibition, promoted differentiation under *PPARγ* control, enhanced mitochondrial ATP synthesis through F_1_F_0_-ATP synthase binding, and reduced oxidative stress (by Figdraw2.0).

Overall, current studies suggest that *G0S2* plays multiple roles in regulating mitochondrial function, apoptosis, and inflammatory responses, but these effects are not uniform and appear to depend strongly on biological context (Figure 2). In some models, *G0S2* has been shown to enhance mitochondrial ATP production and protect cells from oxidative damage, whereas in others it seems to suppress oxidative metabolism and help maintain cellular quiescence. Similarly, both pro-apoptotic and anti-apoptotic actions of *G0S2* have been described. Such discrepancies are likely related to differences in cell type, subcellular localization, upstream signaling, metabolic conditions, and species background. Notably, some of these functions may occur independently of *ATGL*, indicating that *G0S2* should not be regarded solely as an inhibitor of lipolysis, but also as a broader mediator of cellular stress responses. Further clarification of these context-dependent mechanisms will be important for understanding how *G0S2* influences muscle integrity, lipid distribution, and ultimately meat quality in livestock.

## 5. Tissue-Specific Roles of *G0S2* in Lipid Homeostasis

*G0S2* displayed distinct tissue specificity, with high expression in brown adipose tissue (BAT), white adipose tissue (WAT), liver, and skeletal muscle—organs central to lipid metabolism [19]. Moderate expression occurred in kidney and vascular tissues, whereas testes, intestine, and thymus showed minimal levels [41,47,59]. This distribution reflected the differential activity of transcriptional regulators, including *PPARα*, which predominated in liver and BAT, and *PPARγ*, which governed adipogenesis in WAT [41]. During adipogenic differentiation, *G0S2* expression markedly increased but remained unchanged in osteogenic or myogenic pathways, underscoring its role in lipid storage and tissue-specific energy regulation.

### 5.1. Adipose Tissue

In adipose tissue, *G0S2* functioned as a potent inhibitor of lipolysis by specifically inhibiting ATGL activity. Overexpression in adipocytes significantly reduced both basal and hormone-stimulated lipolysis, whereas knockout or knockdown enhanced TG hydrolysis, elevated plasma glycerol, and decreased fat mass [37,60]. *G0S2*-deficient mice exhibited increased lipase activity, elevated expression of thermogenic genes, enhanced lipid oxidation, and improved insulin sensitivity [61]. Conversely, fasting downregulated *G0S2*, facilitating *ATGL*-mediated TG mobilization in pigs [47]. These findings demonstrated that *G0S2* dynamically modulated lipid turnover according to nutritional status. These anti-lipolytic effects reflect altered lipid turnover within existing adipocytes and are mechanistically distinct from the pro-adipogenic role of *G0S2* discussed above. Beyond lipolytic control, *G0S2* was indispensable for adipocyte maturation and lipid accumulation. Its loss in vivo reduced adipose tissue mass and adipocyte size, and overall fat mass, confirming its essential role in fat storage [39]. Transplantation of *G0S2*-deficient adipose tissue lowered plasma TG independently of hepatic very low-density lipoprotein (VLDL) output, suggesting a systemic effect on lipid balance [62]. In muscle-associated adipocytes, *G0S2* further stabilized IMF by restricting *ATGL*-mediated hydrolysis, contributing to IMF retention and thus improving tenderness and juiciness [37]. Its mitochondrial involvement also implied a link between oxidative metabolism and postmortem meat quality. These findings suggest that *G0S2* contributes to adipose expansion through at least two related but mechanistically distinct processes: suppression of triglyceride hydrolysis and facilitation of adipocyte maturation and lipid storage capacity. Collectively, *G0S2* acted as a central modulator of adipose metabolism, balancing lipid mobilization and deposition crucial for meat quality and carcass composition.

### 5.2. Liver

In the liver, *G0S2* regulated both lipid and glucose metabolism. Hepatic *G0S2* deletion diminished TG accumulation, enhanced lipase activity, and improved insulin sensitivity, protecting against diet-induced steatosis [14,60,61,63]. In contrast, overexpression promoted LDs formation and increased circulating TG and LDL/VLDL levels [64,65]. *G0S2* also suppressed PI3K/mTOR signaling, increasing hepatic sensitivity to metabolic stress [44]. These effects established *G0S2* as a key factor linking hepatic lipid flux to systemic energy metabolism. Transcriptionally, *G0S2* was activated by *PPARα*, liver X receptor α (*LXRα*), and *C/EBPβ*. *LXRα* directly bound to a DR4 motif within its promoter to stimulate transcription and hepatic lipid accumulation [66]. Loss of *G0S2* abolished LXR agonist–induced steatosis, defining the *LXRα*–*G0S2* axis as a major determinant of hepatic lipid metabolism [8]. *PPARα* also upregulated *G0S2* during fasting or Wy14643 treatment, though this interaction appeared context-dependent [41,67]. *C/EBPβ* also modulated *G0S2* expression. For instance, in ALK-positive ALCL cell lines, a clear decrease in G0S2 protein levels corresponded to the reduction in C/EBPβ protein [68]. *C/EBPβ* knockdown reduced hepatic lipid storage, while *G0S2* suppression enhanced lipolysis independently of *C/EBPβ* or *PPARγ* levels, indicating that *G0S2* functioned downstream to promote steatosis by inhibiting *ATGL* [25]. Moreover, under endoplasmic reticulum stress, activating transcription factor 4 (ATF4) activated *G0S2* through the PERK–eIF2α–ATF4 pathway, inducing TG accumulation [14]. Together, these data positioned *G0S2* as a central mediator connecting lipid synthesis, storage, and stress responses in hepatocytes. *G0S2* further linked hepatic lipid metabolism to insulin action. Elevated hepatic *G0S2* aggravated insulin resistance by disrupting signaling components, and upregulation of increased *PPARγ* [65], whereas its deficiency enhanced TG breakdown and improved systemic insulin sensitivity [42]. Knockdown also promoted ketogenesis and gluconeogenesis while reducing glycogenolysis [69]. Correlations between *G0S2* and apolipoproteins A4 and A1 suggested additional roles in cholesterol transport [70]. Thus, precise hepatic *G0S2* regulation was critical for maintaining metabolic balance and optimizing energy efficiency. *G0S2* integrated adipose–liver communication by regulating systemic lipid flux. Reduced adipose *G0S2* during fasting enhanced lipolysis and released fatty acids that activated hepatic *PPARα* and *CREBH*, both of which increased hepatic *G0S2* expression to prevent excessive TG breakdown and stabilize energy metabolism [41,42,71,72]. Under chronic lipid overload, however, sustained hepatic *G0S2* activation promoted steatosis and inflammation, indicating its dual metabolic impact.

**Figure 3:**
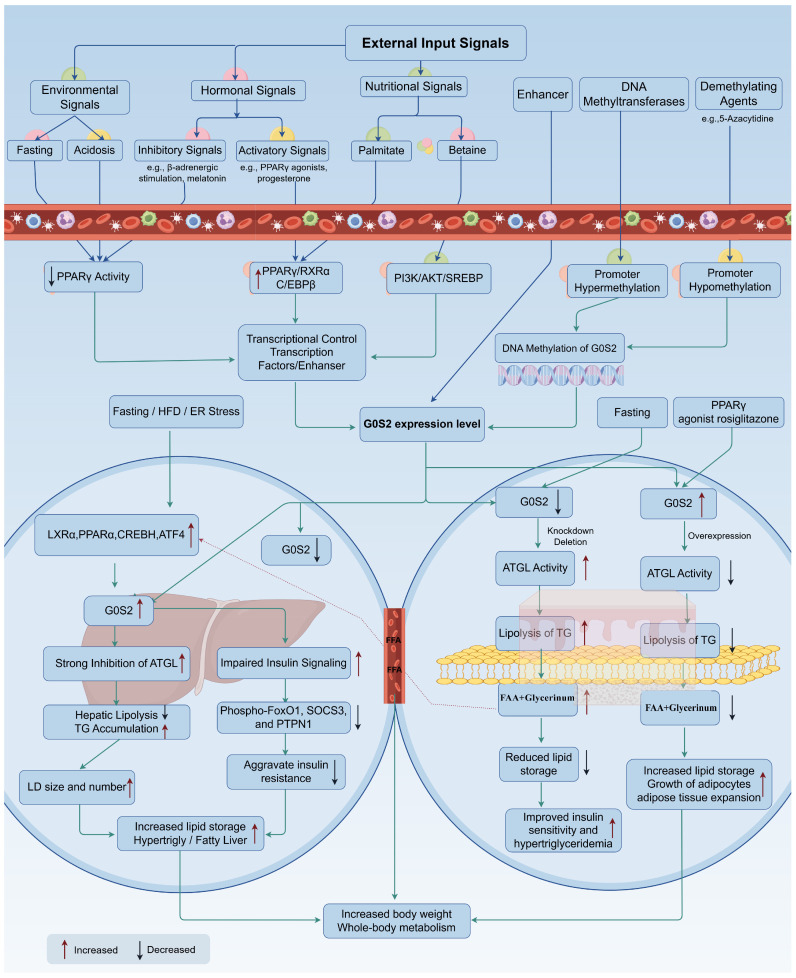
Tissue-Specific Functions and Multifaceted Regulation of *G0S2* Expression by Hormonal, Nutritional, and Environmental Signals. *G0S2* expression was regulated through nutritional status, hormonal cues, and cellular environment. Promoter methylation silenced *G0S2*, while nuclear receptors (*PPARα/γ*, *LXRα*, *FXR*) and stress factors (*CREBH*, *C/EBPβ*) activated it. Functionally, *G0S2* inhibited *ATGL*-mediated lipolysis, promoting TG accumulation in liver and fat storage in adipose tissue (by Figdraw2.0).

Overall, the available evidence supported a tissue-specific role of *G0S2* in adipose tissue, liver, and skeletal muscle (Figure 3). However, the phenotypic outcome was likely influenced by the dominant underlying process, including inhibition of lipolysis, adipocyte differentiation, hepatic triglyceride storage, or muscle-specific metabolic adaptation. Through coordinated transcriptional and endocrine mechanisms, it linked lipid and glucose metabolism, representing a potential target for improving metabolic efficiency and meat quality in livestock.

## 6. Regulatory Factors Controlling *G0S2* Expression

The expression of *G0S2* was tightly regulated by hormonal, nutritional, and environmental factors, reflecting its central role in energy homeostasis and lipid metabolism. It responded dynamically to feeding status, endocrine signals, and stress stimuli, coordinating lipid turnover among adipose tissue, liver, and muscle. Moreover, epigenetic mechanism such as DNA methylation fine-tuned its transcription, influencing tissue-specific functions and metabolic outcomes. Understanding these regulatory layers provided valuable insight into how *G0S2* could be targeted to optimize fat deposition and meat quality in livestock.

### 6.1. Hormonal Regulation

Hormones exerted strong and opposite effects on *G0S2* transcription. Insulin, progesterone, and retinoic acid enhanced its expression, while glucocorticoids, catecholamines, TNF-α, and melatonin suppressed it.

Insulin repressed lipolysis by the PI3K/AKT pathway and stimulated *G0S2* expression in skeletal muscle, consistent with its anabolic role in lipid storage [73,74]. During energy surplus, insulin elevated the *G0S2*/*ATGL* ratio, reducing lipolysis and promoting TG accumulation in adipose tissue [5]. Progesterone acted through its receptor to upregulate *G0S2* and inhibit *ATGL* and *HSL*; these effects were abolished by the antagonist mifepristone [24]. Retinoic acid activated *G0S2* via retinoic acid receptor α (RARα) binding to retinoic acid response elements upstream of the promoter [75]. RAR antagonism or RARα knockdown significantly weakened this induction, demonstrating a direct link between retinoic acid signaling and lipid metabolism via *G0S2*. Similarly, *PPARγ* directly regulated *G0S2* by binding to a PPAR response element about 1.5 kb upstream of its promoter, and its activation by rosiglitazone markedly elevated *G0S2* mRNA during adipocyte differentiation [37,41,76,77].

In contrast, glucocorticoids repressed *G0S2*, thereby activating *ATGL*-mediated lipolysis and increasing plasma NEFA and glucose levels [78]. Catecholamines triggered β-adrenergic and cAMP–PKA signaling, which phosphorylated perilipin A and reduced *G0S2*, facilitating *CGI-58* and *ATGL* recruitment to LDs [5]. In adipocyte models such as 3T3-L1 and SGBS cells, *G0S2* overexpression inhibited adrenergically stimulated lipolysis, while gene silencing enhanced lipolysis and fatty acid efflux [5,37]. Additionally, TNF-α downregulated *G0S2* expression while promoting lipolysis, an effect counteracted by *G0S2* overexpression even under excess *ATGL* or *CGI-58* expression [79]. Melatonin treatment decreased *G0S2* in preadipocytes, upregulated *ATGL*, inhibited adipogenic factors (*C/EBPα*, *PPARγ*), enhanced the expression of fatty acid oxidation-related genes, and mitigated high fat diet induced weight gain in vivo [80]. Thus, *G0S2* functioned as a key regulatory node integrating signals from diverse hormonal and nuclear receptor pathways (*PPARγ*, *PPARα*, *FXR*), fine-tuning lipolysis and adipogenesis according to metabolic and hormonal cues.

### 6.2. Nutritional Regulation

Nutritional status directly modulated *G0S2* expression. Fasting or feed restriction reduced *G0S2* and enhanced *ATGL* in adipose tissue, while refeeding restored basal expression [81]. This fluctuation was coordinated by *PPARα* in liver and *PPARγ* in adipose tissue to redistribute energy during nutritional transitions. Glucose availability was essential for full *G0S2* induction. The ChREBP–Mlx complex, a glucose-responsive regulator, bound to *G0S2* promoter, and *G0S2* mRNA expression was markedly upregulated by glucose in hepatocytes, linking glucose metabolism to lipid storage [82]. Polyunsaturated fatty acids, particularly α-linolenic acid, increased *G0S2* through *PPARγ* activation and suppressed plasma FFA, indicating suppressed lipolysis [83]. In contrast, palmitate induced *G0S2* expression via *C/EBPβ*, accompanied by increased *PPARγ* and downstream adipogenic genes (*G0S2*, *GPR81*, *GPR109A*, and *Adipoq*) in HepG2 cells, promoting TG accumulation by inhibiting *ATGL* [25]. Moreover, dietary betaine attenuated hepatic lipogenesis by suppressing *ACC*, *FAS*, and *SCD1* expression while inducing *G0S2* via the PI3K/AKT/SREBP and *C/EBPα* pathways, resulting in reductions in hepatic cholesterol, bile acid, and glucose levels [84]. Farnesoid X Receptor (FXR) signaling also interacted with c-Jun N-terminal kinase to regulate *G0S2*; its repression by triptolide was attenuated in FXR-null mice, confirming FXR involvement in the liver [85]. Metabolites further exerted cell type–specific effects through *G0S2*. Low concentrations of β-Hydroxybutyric acid downregulated *G0S2* in granulosa cells, inducing G1-phase arrest and impairing cell proliferation [86]. Collectively, *G0S2* functioned as a nutrient-responsive modulator linking dietary signals to cellular lipid metabolism, supporting energy homeostasis. Targeting the nutrient–*G0S2* axis could therefore improve metabolic efficiency and energy balance in animals.

### 6.3. Environmental Stress

Environmental stressors such as oxidative injury and acidosis also altered *G0S2* expression. Oxidized LDL exposure downregulated *G0S2* in endothelial cells, resulting in ROS accumulation, mitochondrial depolarization, cytochrome c release, and caspase-dependent apoptosis. Conversely, *G0S2* overexpression activated AMPK and the Nrf2/SIRT1/HO-1 pathway, reducing oxidative damage [58]. Acidosis promoted lipolysis by disturbing the *ATGL*–*G0S2* balance. In 3T3-L1 preadipocytes, low pH increased *ATGL* while decreasing *G0S2* expression, accelerating lipid hydrolysis. *G0S2* overexpression counteracted this response, confirming its stabilizing effect on LDs [26]. Mechanistically, acidosis suppressed *PPARγ* transcriptional activity; rosiglitazone treatment restored *G0S2* to normal levels. The reduced transcriptional activity of *PPARγ*, possibly caused by impaired interactions with co-activators such as *C/EBPα*, contributed to decreased *G0S2* expression and subsequent ATGL activation. Thus, stress-induced downregulation of *G0S2* disrupted lipid homeostasis, highlighting its role in metabolic adaptation under adverse conditions.

### 6.4. Genetic Regulation

Epigenetic modifications critically shaped tissue-specific *G0S2* expression. Its transcription was regulated by enhancer activity and DNA methylation [87,88]. Multiple enhancers (E2, E4, E5) regulated its transcription, with E5 serving as the major regulatory element bound by *PPARγ* and retinoid X receptor α. Inhibition of E5 reduced *G0S2* and lipogenic genes such as *FABP4*, Lipoprotein Lipase, and *PLIN1*, confirming enhancer-dependent activation [76]. The promoter and exon regions of *G0S2* were CpG-rich, making them prone to methylation by DNA methyltransferases. DNA methylation at CpG-rich promoter regions repressed *G0S2* transcription by blocking transcription factor binding [46,89]. Hypermethylation silenced *G0S2* in various cancers, while demethylation restored expression and inhibited proliferation, whereas knockdown reversed this effect, indicating a tumor-suppressive function [46,90,91]. Promoter methylation also correlated with drug resistance; cisplatin-resistant cells showed repressed *G0S2* due to promoter hypermethylation, whereas sensitive cells expressed it abundantly [92,93]. In immune cells, methyl-CpG binding domain protein 2 enhanced *G0S2* transcription by reducing promoter methylation, facilitating M0-to-M1/M2 transitions. Silencing *G0S2* impaired plasticity, whereas overexpression restored repolarization, indicating that *G0S2* acted as a downstream effector of methyl-CpG binding domain protein 2-mediated macrophage plasticity [94]. Post-translationally, *G0S2* bound *ATGL*, stabilizing both proteins and suppressing lipolysis by preventing ubiquitin-mediated degradation [95]. These epigenetic and protein-stabilizing mechanisms ensured context-specific control of lipid metabolism.

**Figure 4:**
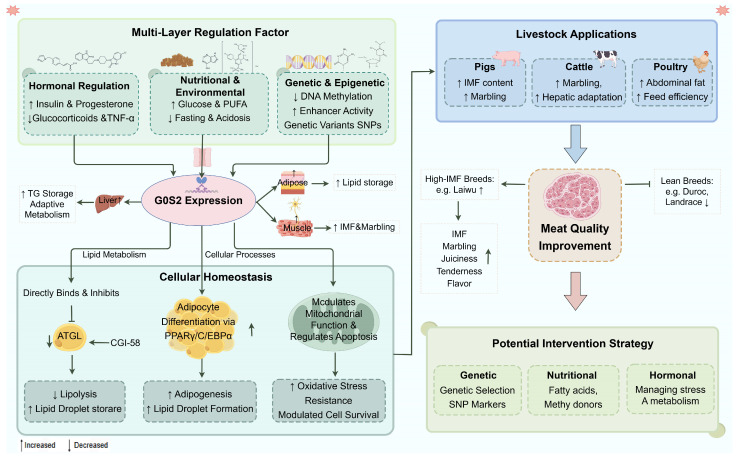
*G0S2*: A central regulator of lipid metabolism and meat quality.

*G0S2* acted as a central hub integrating hormonal, nutritional, environmental, and epigenetic signals to regulate energy storage and mobilization. Hormones such as insulin and progesterone promoted its expression and lipid accumulation, whereas stress hormones suppressed it to enhance lipid mobilization (Figure 4). Nutritional factors and metabolites fine-tuned *G0S2* according to energy status, while environmental stresses and epigenetic modifications further adjusted its expression. Collectively, these mechanisms demonstrated how *G0S2* maintained lipid homeostasis and suggested that manipulating its regulatory network might enhance metabolic efficiency and meat quality in livestock.

## 7. Functional Roles of *G0S2* in Meat Quality and Metabolic

The role of *G0S2* in lipid metabolism had been well established, demonstrating its significant influence on critical economic traits such as fat deposition, meat quality, and feed efficiency.

### 7.1. G0S2 as a Key Regulator of IMF Content in Pigs

In pigs, *G0S2* played a key role in both adipose tissue development and IMF accumulation, two traits critical for carcass value and meat quality. Expression profiling revealed that *G0S2* was abundantly expressed in adipose tissue, liver, and longissimus dorsi muscle tissue, with expression levels increasing during postnatal development as adipocytes mature and lipid content accumulated [47]. Because IMF was a complex trait shaped by the abundance of intramuscular adipocytes, local lipid turnover, and the metabolic characteristics of muscle fibers, the relationship between *G0S2* and pork quality could not be explained solely by inhibition of lipolysis. In longissimus dorsi muscle, higher *G0S2* expression may have favored IMF retention by limiting triglyceride hydrolysis in intramuscular adipocytes, but it may also have reflected differences in adipogenic potential and breed-specific epigenetic regulation. Studies in Chinese indigenous pig breeds further supported this regulatory role. In Laiwu pigs, known for their exceptionally high IMF content (9–12%), *G0S2* was among the lipid biosynthesis genes upregulated during the main phase of IMF deposition (120–240 days), coinciding with changes in DNA methylation [20]. Meta-analyses further identified *G0S2* as a hub gene within a lipid metabolic cluster including *FASN*, *SCD*, and *PLIN1* [21]. The regulatory function of *G0S2* in longissimus dorsi muscle appeared to be species-specific. Comparative analyses revealed that *G0S2* expression was markedly higher in fatty breeds such as Luchuan [96], Songliao Black [21], and Laiwu pigs [20] than in lean commercial breeds including Duroc and Landrace. ATAC-seq data supported these findings, showing enhanced chromatin accessibility in the *G0S2* locus in high-fat breeds, supporting its transcriptional activation and lipid accumulation [96]. However, the upstream regulatory factors and signaling pathways responsible for this species-specific expression, as well as how specific methylation sites dynamically influenced the transcriptional activity of *G0S2*, remained unclear. Genetic polymorphisms within *G0S2*, including Thr89Val (A265G/C266T) and promoter SNPs (g.-565G>A, g.-566T>C), were significantly associated with backfat thickness in several breeds such as Yanan, Jinhua, Duroc, Landrace, and Yorkshire [47,97], highlighting their potential as molecular markers for pork quality selection. Sexual dimorphism was also observed, with sows exhibited higher *G0S2* expression in subcutaneous and perirenal fat, facilitating triglyceride storage through suppression of *ATGL*-mediated lipolysis, whereas boars showed higher expression in visceral organs [97], indicating that sex hormones might be involved in these regulatory mechanisms.

These breed-related differences also interacted with local muscle characteristics, such as oxidative-glycolytic balance, muscle fiber composition, and fiber-associated lipid handling, thereby contributing to variation in IMF deposition and related pork quality traits. The role of *G0S2* in porcine IMF deposition was also interpreted in the context of skeletal muscle fiber characteristics. Pig breeds with higher IMF content were generally associated with a more oxidative muscle phenotype, whereas leaner breeds tended to exhibit a greater proportion of glycolytic fibers. For example, *MyHC I* was positively associated with IMF in Bama Xiang and Rongchang pigs, together with upregulated adipogenic and fatty acid uptake-related genes, including *PPARγ*, *ACC*, *FAS*, and *LPL*, whereas *MyHC IIb* was negatively correlated with IMF in Landrace pigs [98,99]. Moreover, *G0S2* had been identified as a direct target gene of *PPARγ*, further supporting its role in coordinating adipogenic regulation and lipid deposition. These findings suggested that the effects of *G0S2* on IMF deposition depended not only on its anti-lipolytic activity, but also on the local muscle metabolic environment, fiber-type composition, and related regulatory networks, such as AMPK and PGC-1α signaling.

Taken together, these findings supported the view that *G0S2* was a central regulator of porcine IMF deposition through multiple interacting mechanisms. On the one hand, *G0S2* may have promoted lipid retention by inhibiting *ATGL*-mediated lipolysis in intramuscular adipocytes, while the *PPARγ*–*G0S2* axis appeared to be a plausible regulatory pathway contributing to porcine IMF deposition. on the other hand, its phenotypic effects may also have been influenced by breed-specific chromatin accessibility, DNA methylation, developmental stage, sex-dependent regulation, and the metabolic properties of skeletal muscle. Thus, the relationship between *G0S2* expression and pork quality should not have been interpreted solely as a simple consequence of reduced lipolysis. Rather, it was more likely to reflect the combined effects of lipid turnover, adipogenic capacity, and muscle-specific metabolic adaptation. These observations highlighted *G0S2* as a promising candidate gene for improving pork quality, while also indicating that its precise regulatory mechanism, particularly its interaction with the muscle microenvironment and upstream metabolic signaling pathways, requires further investigation.

### 7.2. G0S2 as a Regulator of Marbling and Hepatic Metabolic Adaptation in Cattle

In cattle, *G0S2* was regarded as an important regulator of intramuscular fat deposition, marbling, and hepatic energy adaptation. It was preferentially expressed in adipose tissue and skeletal muscle, with moderate expression in the liver. In longissimus dorsi, *G0S2* expression was positively correlated with IMF content [22]. Steers showed higher *G0S2* expression than bulls, which was consistent with the greater IMF accumulation observed after castration, together with the upregulation of *FABP4* and *CGI-58* [22]. Notably, *G0S2* was enriched in the IMF fraction, whereas *CGI-58* was more abundant in muscle fibers, suggesting that *G0S2* was more closely associated with adipocyte-related lipid storage within the intramuscular compartment than with intramyocellular triglyceride metabolism itself. This distinction was important because marbling primarily reflects adipocyte-associated lipid deposition, whereas intramyocellular lipid pools were more tightly linked to fiber metabolism and oxidative activity. By suppressing lipolysis in intramuscular adipocytes, *G0S2* was thought to favor IMF accumulation and thereby contribute to meat quality traits such as juiciness and flavor. In Mongolian cattle, *G0S2* participated in adipogenesis by promoting the progression from preadipocyte commitment to mature adipocyte formation and by influencing endocrine and energy storage functions [100]. Beyond adipose regulation, hepatic *G0S2* expression increased in early-lactating dairy cows in parallel with genes involved in lipid and cholesterol secretion, including apolipoprotein A1 and apolipoprotein A4, and this response was likely regulated by the insulin receptor, *PPARγ*, and peroxisome proliferator-activated receptor gamma coactivator 1-alpha [70,101]. This upregulation was considered an adaptive response to postpartum energy demands, helping to stabilize hepatic triglyceride levels and maintain energy balance. Altogether, current data indicate that *G0S2* supports beef marbling mainly through regulation of intramuscular adipocytes, while also contributing to hepatic metabolic adaptation in dairy cattle. However, the final phenotype is likely modified by muscle fiber composition, local metabolic activity, endocrine status, and stage of production, current evidence does not yet fully resolve the relative contribution of these distinct mechanistic processes.

### 7.3. G0S2 as a Central Regulator of Lipid Metabolism in Poultry

Although poultry meat contained relatively low IMF, *G0S2* remained a critical regulator of lipid metabolism, influencing abdominal fat deposition, feed efficiency, and immune function. Avian *G0S2* shared a conserved hydrophobic domain with mammalian orthologs and was mainly expressed in adipocytes, where it inhibited *ATGL* to limit lipolysis [81]. Expression increased during adipocyte differentiation, suggesting a function in terminal differentiation rather than proliferation. Sex-, age-, and tissue-specific differences were observed: abdominal fat showed higher expression in females at 21 and 91 days, while hepatic expression was higher in males and declined with age [23]. Several single nucleotide polymorphisms (SNPs), including G102A, G197A, G255A, g.444G>A, g.102G>A, and g.556G>A, were significantly associated with abdominal fat and body weight [23,102]. The g.444G>A variant correlated with abdominal fat weight, whereas AA/AG genotypes of g.102G>A were linked to increased live weight. Knockout studies confirmed that loss of *G0S2* reduced abdominal fat, altered TG synthesis, and changed fatty acid composition without affecting growth performance, consistent with its role in regulating lipolysis [103]. Quail models further demonstrated that *G0S2* overexpression suppressed fasting-induced lipolysis [104]. Differential *G0S2* expression between high- and low-feed-efficiency groups, likely regulated by intron retention affecting mRNA stability, indicated a role in lipid partitioning and energy utilization [105]. Additionally, *G0S2* expression was inducible during Salmonella Enteritidis infection, suggesting a protective function by limiting lipid oxidation and reducing ROS-mediated tissue damage [11,106]. These findings demonstrated that *G0S2* served as a core regulator of lipid metabolism in poultry, influencing fat deposition, energy efficiency, and immune response.

Overall, *G0S2* functions as a conserved but context-sensitive regulator of lipid metabolism across species. Elevated *G0S2* expression is often associated with higher IMF, improved marbling, or increased fat deposition (Table 1), but these associations did not necessarily arise from the same mechanism in every tissue or species. Its expression is modulated by genetic variants, epigenetic regulation, and environmental cues. Given these characteristics, *G0S2* represents a promising molecular target for marker-assisted selection and nutrition-based interventions aimed at improving meat quality and production efficiency.

## 8. Conclusions

*G0S2* was recognized as a conserved and multifunctional regulator of lipid metabolism, energy balance, and cellular homeostasis, with potential relevance to livestock meat quality and production efficiency (Figure 4). Its canonical role as an endogenous inhibitor of ATGL provided a clear mechanistic basis for limiting triglyceride hydrolysis and favoring lipid retention. However, accumulating evidence indicated that the biological function of *G0S2* extended beyond lipolysis and included adipocyte differentiation, mitochondrial regulation, apoptosis, and inflammatory signaling. These functions suggested that *G0S2* contributed to tissue lipid accretion through multiple pathways rather than through a single mechanism alone. The consequences of *G0S2* expression vary with tissue type, cell lineage, metabolic state, and local muscle environment, and may reflect distinct contributions of reduced lipolysis, enhanced adipocyte differentiation, mitochondrial adaptation, and inflammatory or apoptotic signaling. In livestock species, *G0S2* expression was closely associated with fat deposition patterns, IMF content, and marbling, key determinants of tenderness, juiciness, and flavor. Studies in pigs, cattle, and poultry consistently showed that upregulated *G0S2* promoted lipid accumulation, while its suppression enhanced lipolysis and reduced IMF deposition. These findings established *G0S2* as a pivotal molecular switch linking intracellular lipid metabolism to economically important meat quality traits. Thus, *G0S2* should be viewed as an important but context-dependent regulator linking intracellular lipid metabolism to economically relevant meat-quality traits. The regulation of *G0S2* expression appeared to be multifactorial and tightly controlled by hormonal, nutritional, and environmental factors. Insulin and *PPARγ* agonists induced its transcription, whereas glucocorticoids and catecholamines exerted inhibitory effects. In addition, nutrient status and epigenetic modifications further influenced its expression, highlighting the gene’s responsiveness to both physiological and environmental cues. Collectively, these findings positioned *G0S2* as a promising molecular target for enhancing livestock meat quality trait through coordinated genetic, nutritional, or hormonal interventions.

Future research will clarify species- and tissue-specific mechanisms of *G0S2* using advanced genetic models, including CRISPR/Cas9-mediated *G0S2* knockout or overexpression animals. Integration of multi-omics technologies (Genomics, Transcriptomics, DNA methylation, and Lipidomics) with refined phenotyping will be essential to reveal regulatory networks linking *G0S2* activity to IMF, marbling, fatty acid profiles, and sensory traits. Future work should also explicitly distinguish intramuscular adipocyte deposition from intramyocellular lipid accumulation and incorporate muscle fiber typing, and local oxidative-glycolytic status, when evaluating *G0S2*-associated marbling phenotypes. Modulating *G0S2* expression through dietary fatty acids, methyl donors, or feed additives may provide practical strategies for optimizing IMF without increasing total carcass fat. Overall, a comprehensive understanding of its pleiotropic roles can facilitate the development of biomarker-based selection and nutritional interventions, ultimately contributing to improve meat quality and sustainable livestock production.

## Figures and Tables

**Table 1:** Comparative summary of studies on *G0S2* gene functions across species.

Species	Primary Role in Meat Quality	Results	Reference
Pig	Regulator of IMF deposition	Dynamic transcriptome and methylome analyses revealed *G0S2* as a potential key gene influencing IMF content.	Wang et al., 2017 [20]
	Regulator of IMF deposition	Meta-analysis of porcine muscle transcriptomes identified *G0S2* among major regulators of IMF deposition.	Wang et al., 2025 [21]
	Modulator of muscle metabolism and adipogenesis	ATAC-seq and RNA-seq analysis identified *G0S2* as a muscle development and lipid metabolism candidate gene, suggesting active regulation in muscle.	Miao et al., 2021 [96]
	Genetic marker for lipid storage	*G0S2* showed differential expression among tissues, and the adipose tissue expresses at a higher level. Polymorphisms were associated with fat-related traits (backfat thickness and carcass length).	Jiang et al., 2014 [99]
	Regulation of adipogenesis and IMF deposition	*G0S2* expression was dynamically regulated during adipogenesis and short-term nutritional interventions, suggesting its involvement in lipid metabolism and IMF accumulation.	Ahn et al., 2013 [47]
Cattle	Association with IMF and energy metabolism	Differential expression of *G0S2* in high- and low-marbled muscle was correlated with IMF content and lipid turnover in bovine muscle.	Ahn et al., 2014 [22]
	Adipose tissue cellular response to environmental stress	snRNA-seq revealed *G0S2*-mediated lipid and immune regulation under cold and disease resistance conditions.	Chi et al., 2024 [100]
	Hepatic transcriptomic response to metabolic stress	The liver transcriptome showed *G0S2*-related changes depending on postpartum subacute ruminal acidosis occurrence, and response to periparturient metabolic stress.	Tsuchiya et al., 2021 [101]
	Liver metabolic adaptation during transition period	*G0S2*-related pathways were involved in metabolic adaptation around parturition in dairy cows, which might be related to the excessive energy mobilization.	Ha et al., 2017 [70]
	Nutritional regulation of gluconeogenesis	Liver metabolic adaptation during transition period	Zhang et al., 2015 [69]
Poultry	Direct inhibitor of fat deposition	*G0S2* disruption reduced abdominal fat deposition and altered fatty acid composition in muscle, confirming its role as a key lipolytic inhibitor in vivo.	Park et al., 2019 [103]
	Inhibitor of lipolysis	*G0S2* overexpression inhibited lipolysis in adipose tissue during feed restriction in transgenic quail, demonstrating inhibition of lipolysis and resistance to loss of fat by *G0S2*.	Shin et al., 2014 [104]
	Regulator of adipose tissue development	*G0S2* expression was upregulated during adipogenesis and influenced by developmentally and nutritionally regulated, indicating a key role in controlling fat mass in chicken adipose tissue.	Oh et al., 2011 [81]
	Relationship with carcass traits	Specific polymorphisms in *G0S2* were significantly associated with carcass traits, suggesting its use for marker-assisted selection.	Yang et al., 2022 [102]
	Gene cloning and association with production traits	*G0S2* cDNA was cloned; expression and polymorphisms were associated with growth and carcass traits (abdominal fat deposition).	Zeng et al., 2011 [23]
	Post-transcriptional fat regulation and feed efficiency	3′UTR-seq revealed intron retention and *G0S2* involvement in fat deposition and feed efficiency regulation.	Wang et al., 2022 [105]

## Data Availability

No new data were created or analyzed in this study.

## Abbreviation

ATF4, activating transcription factor 4; ATGL, adipose triglyceride lipase; BAT, brown adipose tissue; C/EBPα, CCAAT/enhancer-binding protein α; C/EBPβ, CCAAT/enhancer-binding protein β; CGI-58, comparative gene identification-58; CREBH, c-AMP responsive element binding protein H; FASN, Fatty Acid Synthase; FFA, free fatty acids; G0S2, G0/G1 switch gene 2; HSL, hormone-sensitive lipase; LXRα, liver X receptor α; OXPHOS, oxidative phosphorylation; PLIN1, perilipin-1; PKA, protein kinase A; PPARα, peroxisome proliferator-activated receptor α; PPARγ, peroxisome proliferator-activated receptor γ; PNPLA, patatin-like phospholipase domain-containing protein; ROS, reactive oxygen species; TG, triglycerides; TNF-α, Tumor necrosis factor-alpha; VLDL, hepatic very low-density lipoprotein; WAT, white adipose tissue.
